# A straightforward microfluidic-based approach toward optimizing transduction efficiency of HIV-1-derived lentiviral vectors in BCP-ALL cells

**DOI:** 10.1016/j.btre.2023.e00792

**Published:** 2023-03-12

**Authors:** Seyed Esmaeil Ahmadi, Rima Manafi Shabestari, Amir Asri kojabad, Majid Safa

**Affiliations:** Department of Hematology and Blood Banking, Faculty of Allied Medicine, Iran University of Medical Sciences, Tehran, Iran

**Keywords:** Lentiviral vector, Microfluidics, Transduction efficiency, Acute lymphoblastic leukemia, LVs, HIV-1-derived lentiviral vectors, BCP-ALL, B-cell precursor acute lymphocytic leukemia, mm, millimeters, cm, centimeters, μm, micrometers, MFS, microfluidic systems, VSV-G, vesicular-stomatitis-virus-G glycoprotein

## Abstract

**Background:**

HIV-1-derived lentiviral vectors (LVs) are capable of transducing human cells by integrating the transgene into the host genome. In order to do that, LVs should have enough time and space to interact with the surface of the target cells. Herein, we used a microfluidic system to facilitate the transduction of BCP-ALL cells.

**Methods and Results:**

We used a SU-8 mold to fabricate a PDMS microfluidic chip containing three channels with a 50 μm height and a surface matching 96-well plates. In order to produce LVs, we used HEK293T cells to package the second generation of LVs. First, we evaluated the cell recovery from the microfluidic chip. Cell recovery assessment showcased that 3 h and 6 h of incubation in microfluidic channels containing 100,000 NALM-6 (BCP-ALL) cells with 2μL of culture media yielded 87±7.2% and 80.6 ± 10% of cell recovery, respectively. Afterward, the effects of LV-induced toxicity were evaluated using 10–30% LV concentrations in time frames ranging from 3 h to 24 h. In 96-well plates, it took 12–24 h for the viruses with 20% and 30% concentrations to affect the cell survival significantly. These effects were intensified in the microfluidic system implying that microfluidic is capable of enhancing LV transduction. Based on the evidence of cell recovery and cell survival we chose 6 h of incubation with 20% LV.

**Conclusion:**

The results from EGFP expression showcased that a microfluidic system could increase the LV transduction in BCP-ALL cells by almost 9-folds. All in all, the microfluidic system seems to be a great armamentarium in optimizing LV-based transduction.

## Highlights

• Lentiviral vectors have a limited half-life, and in order to reach the target, the distance between the target cell and the vector should be minimized.
• Microfluidic systems provide a micro-scaled environment in which lentiviral vectors have a high chance of reaching the target cell.
• Using microfluidic systems decreases the transduction time.
• Compared to standard cell culture plates, microfluidic systems require considerably lower culture media and reagents for a transduction procedure.
• Microfluidic system has proven to be very beneficial in enhancing lentiviral transduction.

## Graphical abstract

**Figure:**
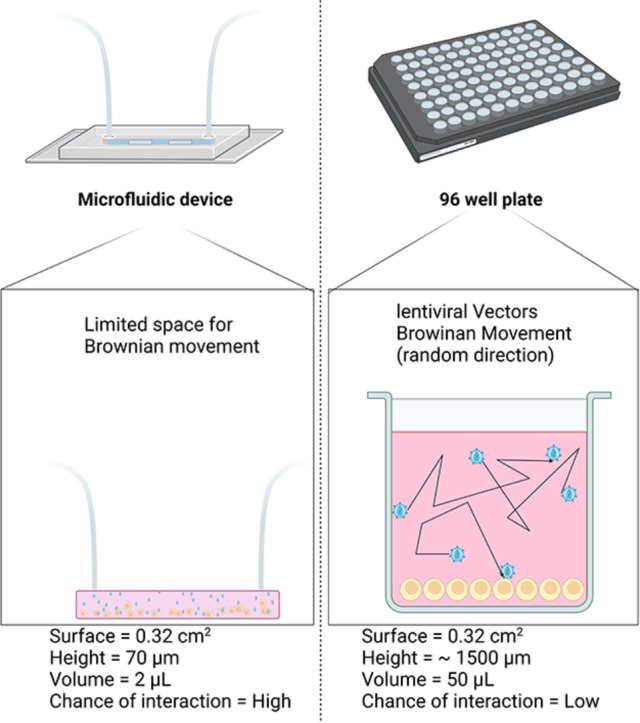

## 1 Introduction

Human B lymphocytes are attractive targets to be transduced with HIV-1-derived lentiviral vectors (LVs) for basic research and gene therapy [1]. However, B cells seem to be relatively hard to transduce with LVs [2,3]. Manipulating signaling pathways is an excellent way to comprehend the cell's machinery in order to find a potential factor for targeted-therapy purposes or find the underlying reasons for disease development. Therefore, to alter these pathways, LVs can be used as a great armamentarium. Besides, B-cells potential for antigen presentation and antibody production also makes them intriguing targets for LV-mediated integration of desired transgenes [2].

Three generations of pseudotyped replication-deficient LVs have been developed for different purposes, such as gene therapy and basic research [4,5]. However, the first generation of LVs has been put aside due to the existence of almost all HIV-1 accessory genes (gag, pol, env, Tat, Rev, Vif, Vpr, Vpu/Vpx, Nef) in the vector system [6]; consequently, there is a risk of generating replication-competent LVs. In the second generation, the accessory genes not crucial for LV production have been removed in order to increase the biosafety of the vectors, though it seems not to suffice for clinical use. In contrast, the third generation does not contain a gene called Tat; and the Rev gene is encoded by a distinct plasmid, and 5՛LTR has been replaced by chimeric LTR, which all can provide a clinical-grade LV [4,5]. It is worth mentioning that compared to the second generation, the third generation provides a lower titer of vector due to the aforementioned changes, although there is no significant difference between these generations regarding transduction efficacy [7,8]. LVs can infect both dividing and non-dividing cells, privileging them to infect various immune cells, in which primary B cells have been proven to be hard to transduce [3], though cancerous B cells seem easier targets for LVs. Herein, we used NALM-6 cell line as the representative of B-cell precursor acute lymphocytic leukemia (BCP-ALL).

Many approaches have been taken into account to enhance LV transduction efficiency, including polycations (e.g., polybrene and protamine sulfate) [9], spinoculation [10], Retronectin [11], and some adjuvants (e.g., Rapamycin [12], Dexamethasone [13], BX795 [14], Cyclosporine A and H [15], Staurosporine [16], phorbol 12-myristate13-acetate (PMA) [17], prostaglandin E2 and Poloxamer 407 [18]). The abovementioned approaches have been used in standard transduction systems, such as well-plates and flasks. LVs require to move distances in millimeters (mm) or centimeters (cm) in the standard platforms to attach to the target cells. Noteworthy that in standard cell culture systems at 37 °C, LVs can travel approximately 600 micrometers (μm) via the Brownian movement (pedesis) with a half-life in a matter of hours. LVs do not predictably traverse distances in this type of movement; instead, they move and fluctuate randomly across the culture media. Usually, dealing with this challenge, requires using a relatively high volume of LVs and/or multiple rounds of transduction to get the viruses to the cells [19,20]. The ratio of viral vectors to target cells referred to as MOI is a conventional index for vector dosage determination in transduction. However, it has been declared that MOI is unreliable for determining LV dosage [21,22]. On the other hand, vector concentration by volume could be used as the alternative index to represent the maximum LV particles required for a non-cytotoxic transduction procedure [23,24]. In order to concentrate the viral particles, methods such as ultracentrifugation, gradient-based centrifugation and ultrafiltration could be utilized [25,26], which all has their limitations regarding the recovery of vector particles.

**Fig. 1:**
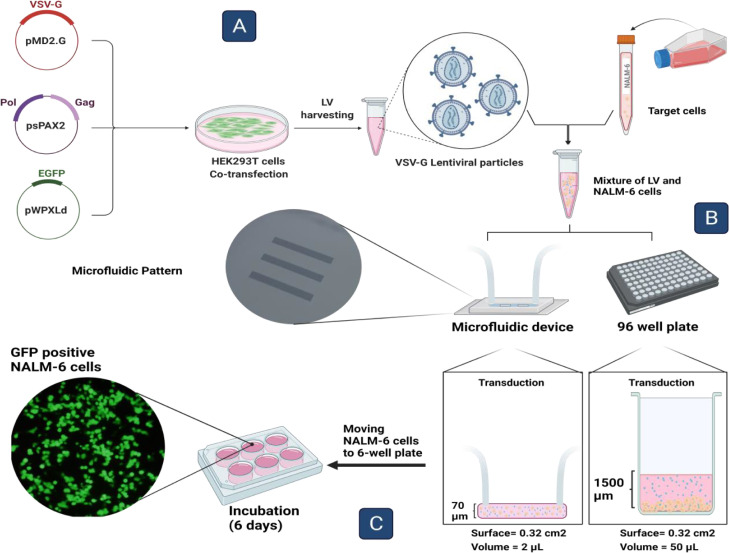
Schematic of LV transduction process. **A**: HEK293T cells are transfected with a second generation of LV plasmids encoding EGFP transgene, which eventually produce LV-GFP. **B**: harvested LVs mixed with the target cells (NALM-6) would be transferred into 96-well plates and MFS to allow the LVs to transduce the cells. The main difference between MFS and 96-well plates is the considerably low height provided by MFS while maintaining a surface equal to 96-well plates. Since LVs' movement is unpredictable and their half-live is limited, many of LVs could be wasted in standard culture systems. **C**: After incubation in both platforms, the LV particles should be removed from NALM-6 cells and they are transferred to a bigger culture plate to expand and express the EGFP protein.

Two mentioned limitations in the way of efficient LV transduction, including a limited spacial traverse of LVs considering their half-life in conventional culture media and providing practical, nontoxic doses of LVs, could be addressed by using microfluidic systems (MFS), which offer a dynamic micro-scaled environment (Fig. 1). MFS can readily provide a surface area equal to 96-, 48-, 12- and 6-well plates with a micro-scaled height [27]. Putting cells and LVs in a micro-scaled environment over a large surface area like standard culture plates eliminates the limitations regarding spatial distances and the use of high LV concentrations, which could be toxic [27].

In this study, we implemented a straightforward microfluidic-based approach toward enhancing transduction of vesicular-stomatitis-virus-G glycoprotein (VSV-G) pseudotyped LVs encoding an EGFP transgene in a BCP-ALL derived cells, NALM-6. A head-to-head analysis of transduction efficiency in MFS and 96 well-plates was performed, where the LV concentrations (v/v%), transduction times, cell survival, and surface-to-volume ratio for optimum transduction were evaluated. Standard transduction platforms such as 96-well plates require a relatively high volume of media and reagents, where a considerable amount of LV particles could be wasted due to the height of fluids (Fig. 1). In this manner, we used the lowest volume of media (50 μL) possible for proper transduction in 96 well-plates, providing the lowest surface-to-volume ratio for optimum transduction with a ∼1500 μm fluid height. In this study, to optimize LV transduction of NALM-6 cells, we utilized a micro-scale cell culture platform in which the working volume (∼2 μL) was low while the surface area mirrored a 96-well plate. Only a single round of transduction was applied for both platforms. Since we minimized the volume (2 μL) and height (70 μm) with a microfluidic chip, we did not concentrate the viral vectors using methods like ultracentrifuge in order to perform the transduction as straightforwardly as possible.

## 2 Materials and methods

### 2.1 Cell culture

Prior to transfection, HEK 293T (ATCC CRL-3216) cells were cultured in high glucose Dulbecco's modified Eagle's medium (DMEM; Gibco), supplemented with 10% fetal bovine serum (FBS) (BioChrom), 1% l-glutamine and 1% of Pen/Strep (10,000 units/mL penicillin and 10,000 µg/mL streptomycin). Prior to transduction, NALM-6 cells (ATCC CRL-3273) were cultured in RPMI 1640 (Gibco) media supplemented with 10% FBS, 1% l-glutamine and 1% 1X-Pen/Strep. All cells were incubated at 37 °C at 5% CO_2_.

### 2.2 Microfluidic fabrication

**Fig. 2:**
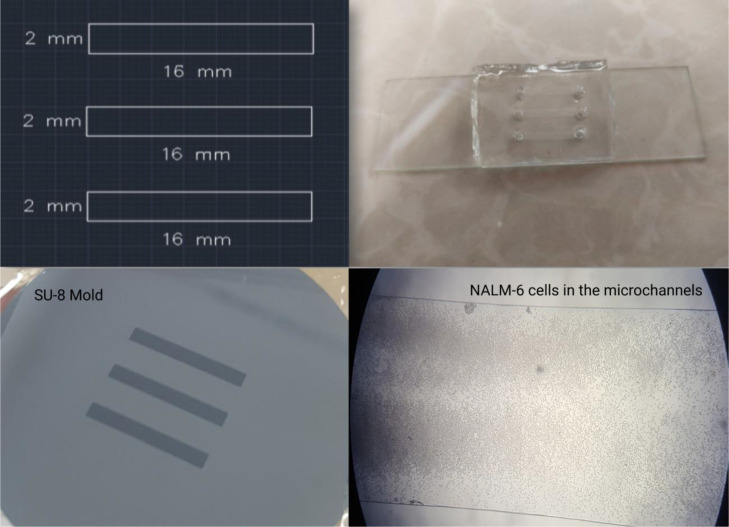
Details of microfluidic system used for LV transduction. Based on the length, width and height of the channels (∼70 μm), the volume of each channel is ∼2 μL. Also, the presence of NALM-6 cells in the microchannel has been shown.

In the first step of making a microfluidic chip, a photomask by CAD software was designed. To prepare the microfluidic mold, the epoxy-based negative photoresist SU-8 2050 (Kayaku Advanced Materials) was spincoated on a 2-inch silicon wafer to attain 70 μm height, and then it underwent soft baking for 5 min at 65 °C followed by 20 min at 95 °C. The photomask with the desired pattern was placed on the photoresist-coated silicon, and UV exposure led the pattern to crosslink into the SU-8. The developer solution was used to remove the non-crosslinked SU-8. Afterward, Polydimethylsiloxane called PDMS (Sylgard 184; Dow Corning) mixed with the curing agent (Mix ratio 10:1) was cast on the mold. The mold was left for 1 h to remove bubbles, and then it was baked at 65 °C for 3–4 h. Once it was cured, a rectangular piece of PDMS containing our channels was cut, and prior to adhesion on the glass slide, a biopsy punch (2 mm) was used to insert holes for inlets and outlets. Corona plasma treatment gun (BD-20AC, Electro-Technic Products, IL, USA) was used for 50 s to create an adhesive surface on the glass slide and the crafted PDMS. After adhesion, tubes were placed into the device, and flushing the channels with culture media ensured that there was no obstruction or leakage. Our chip contained three channels, each with a 0.32 cm^2^ surface area equal to a well in the 96-well plate (Fig. 2). Considering the ∼70 μm height of the channels, the volume of each channel was ∼2 μL.

### 2.3 Lentiviral vector production

HEK 293T cells (5 × 10^6^) were cultured in a 6 cm culture plate to reach a 70–80% confluency. Afterward, 4 h before the transfection, the media was replaced with a serum-free DMEM. A second-generation lentiviral vector system was used in this study, in which the backbone EGFP encoding plasmid (pWPXLd), alongside plasmids for packaging (psPAX2) and VSV-G envelope (pMD2.G), were mixed in a 4:4:1 ratio. To prepare the transfection reagent (TR), 15 μL of PolyFect transfection reagent (Qiagen) was mixed with 170 μL serum-free DMEM. The mixture of plasmids and TR solution was incubated at room temperature for 20 min and then added dropwise to the HEK 293T cells. The culture plate was returned to the incubator (37 °C, 5% CO_2_). After 16 h, the media was changed with a DMEM containing 5% FBS. Seventy-two hours post-transfection, the supernatant containing viral particles was collected (Fig. 1). To remove debris, it was centrifuged (1500 rpm, 10 min, 4 °C), and filtered using a 0.45 μm pore PVDF Millex-HV filter (Millipore). The presence of LV particles in the viral stock was assessed by an ELISA-p24 kit (Pishtaz Teb).

### 2.4 Lentiviral transduction

NALM-6 cells were centrifuged at 1500 rpm for 10 min to remove growth media. The cells were resuspended in RPMI 1640 media containing 4 μg/mL polybrene and LV. Noteworthy that the ratio of the LV volume added to the mixture should not exceed 30% [28]. Transductions in MFS and well plate controls were run concurrently, applying equal LV concentrations (%10,%20, and 30%) in identical transduction times (3, 6, 12 h) upon 100,000 NALM-6 cells. Prior to pumping the cells into MFS, channels were washed with 70% ethanol, followed by RPMI. The cells were gently pumped into the channels at 0.5 μL/min speed, which took 4 min to fill the channel. It should be mentioned that following filling microchannels, the flow was disconnected, allowing the viruses to infect the target cells. Transduction in the 96-well plate was conducted with a total volume 50 μL. Well, plates and microfluidic devices were incubated for 12 h at 37 °C at 5% CO_2_. Post-infection, cells were flushed out from MFS via pumping 2 mL of culture media through channels into 15 ml Falcon tubes. The tubes were centrifuged at 1500 rpm for 10 min to remove the vector-containing media. Afterward, cells were resuspended in a growth media and were transferred to a 6-well plate for expansion. The same was conducted for the cells recovered from the 96-well plate.

### 2.5 Assessment of cell recovery and cell survival

In this process, cultured cells in microfluidic and 96 well-plates were collected and transferred to a microtube to be centrifuged (1500 rpm, 10 min). Finally, the resuspended cell pellets in fresh media were counted using the trypan blue staining technique [29], showcasing the number of cells that remained alive. This step is necessary since environmental factors, media conditions, flushing the cells into microchannels, and the virus itself might affect the viability of the cells. More precisely, it should be mentioned that cell recovery and cell survival are the same processes, with different stress levels upon cells. In cell recovery, we assess the survival of cells under environmental stresses, especially in MFS, without the presence of viral vectors. Afterward, cell survival is the next step, and the toxicity of the viral vector will be added to the equation.

### 2.6 Assessment of GFP expression

Cells were maintained and cared in culture media for six days post-transduction, and then cells in each well were assessed by fluorescence microscope (Nikon, Eclipse Ts2R) and BD FACSCalibur Flow Cytometer.

### 2.7 Statistical analysis

GraphPad Prism 8 was used to analyze the data. All data are represented as means ± SD. The statistical significance was determined by the Students *t*-test and groups were compared using two-way ANOVA. A p-value of <0.05 was considered to be significant.

## 3 Results

### 3.1 Cell recovery

**Fig. 3:**
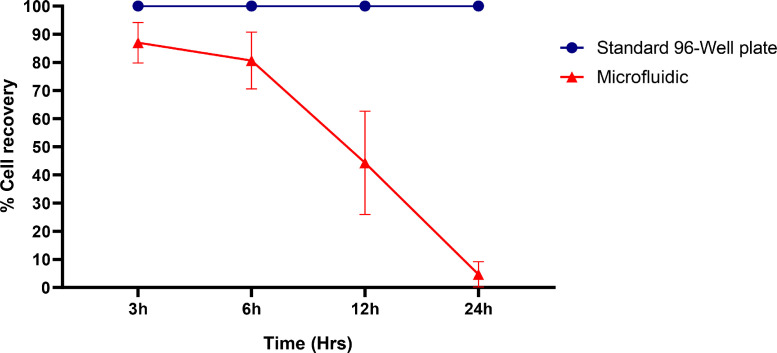
Environmental tensions upon cells during incubation can affect cell recovery, and in micro-scaled environments, these tensions could be intensified; so, the cell recovery should be carefully measured. In 3 h group cell recovery in MFS was 87±7.2% and decreased to 80.6 ± 10% and 44.3 ± 18.3% in 6 h and 12 h groups, respectively. Due to the environmental tensions in MFS, almost all cells were dead in 24 h group. Based on evidences 6 h were chosen as the proper incubation time for transduction.

Due to excessive tension on cells in micro-environment conditions, considering limited nutrients and media conditions, cell recovery is accounted for a crucial issue. We compared cell culture in 96-well plate and microfluidic channels by adding 50 μL and 2 μL media containing 100,000 cells in each microfluidic channel and 96 well-plates followed by incubation for 3, 6, 12 and 24 h (Fig. 3). After centrifuging and media exchanging, almost all of the cells from 96 well-plates were recovered alive in all time frames. On the other hand, in the microfluidic system, we recovered approximately 85%, 80%, 44%, and 4% of the cells alive after 3, 6, 12 and 24 h, respectively. In MFS, the longer the incubation, the more cells would be lost, which could mostly result from environmental tensions. Since more than 6 h of incubation significantly affects the viability of the cells in our microfluidic device, we chose this time frame as the optimal time for incubation.

### 3.2 Viral toxicity and cell survival

**Fig. 4:**
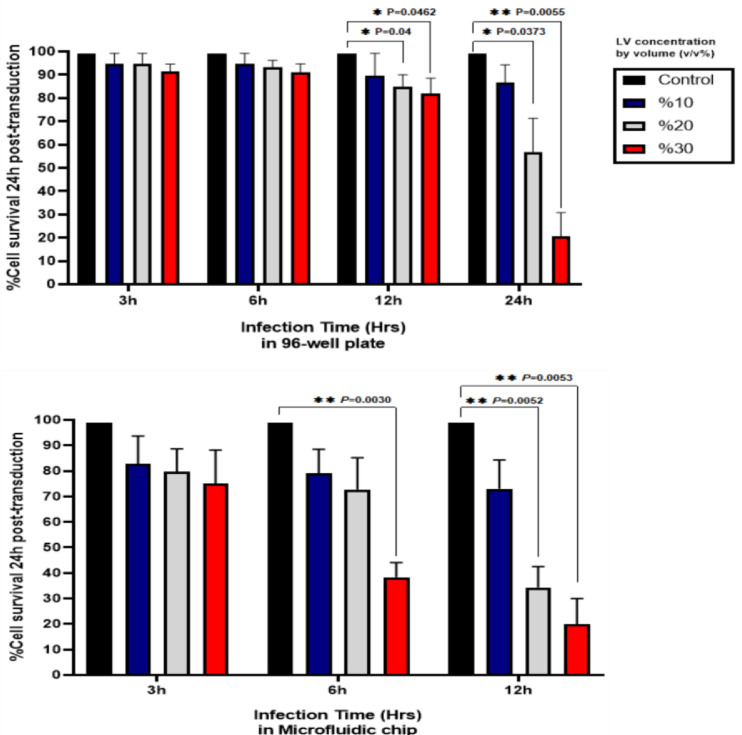
To assess the effects of LV-induced toxicity alongside other environmental tensions on NALM-6 cells, the vectors were introduced to the cells in 10–30% concentrations in different time frames. In the 3 h and 6 h groups of 96-well plates, LVs effects on the cells were not significant. However, cell survival significantly decreased in 12 h and 24 h groups, especially with 20% and 30% of LV concentrations. Based on evidence 6 h of incubation with 20% LV concentration seemed suitable for proper transduction.

After assessing the cell recovery, a proper transduction approach was set where the cells needed to be infected with viral particles in both microfluidic devices and 96 well-plates to measure the effects of viral particles. Following infecting the NALM-6 cells in both platforms for 3 to 24 h, trypan blue staining revealed that a portion of the cells were dead 24 h post-infection, which could be due to the virus-induced toxicity. The effects of viral toxicity on NALM-6 survival were evaluated using different LV concentrations by volume (v/v%) in multi-time frames (Table 1). The cells were exposed to 10%, 20%, and 30% concentrations of viral particles in time periods of 3, 6, 12 and 24 h. However, since in MFS, the cell recovery of cells infected with virus for 24 h was less than 5% we removed this time period for this group (Fig. 4). Following the transduction step, the cells were transferred to a 6 well-plate with fresh media for expansion and after 24 h the cell survival was assessed. Of note, increasing transduction time could intensify the viral toxicity and lower the viability of cells.

**Table 1:** Post-transduction cell survival.

Transduction system	Transduction time frames		Survival (Mean±SD) (*n* = 3)		
			**10%**	**20%**	**30%**
Microfluidic System		3h	82.66±11	79.66±9.018	75±13.22
		6h	79±9.53	72.66±12.50	38.33±5.77
		12h	73±11.26	34.33±8.14	20±10
96-well plates		3h	94.66±4.50	94.66±4.50	91.33±3.21
		6h	94.66±4.50	93.33±2.88	91±3.60
		12h	89.66±9.50	85±5	82±6.55
		24h	86.66±7.63	56.66±14.57	20.66±10.06

In MFS, 24 h post-infection, the cells in all viral concentrations of 3 h groups showed 75% to 82% cell survival. This indicated that even increasing the LV concentration in this time period did not have a significant effect on the NALM-6 cells. In 6 h group, 79% and 72% of the cells remained alive following exposure to the 10% and 20% LV concentrations, respectively, although increasing the concentration of LV to 30% caused a significant cell death, in which only 38% of cells was alive 24 h post-infection. Cell survival significantly decreased in 12 h group, especially with 20% and 30% LV concentrations, in which only 34% and 20% of the cells survived, respectively. Intriguingly, the recovered cells from 12 h group with 10% LV concentration had 73% cell survival after 24 h, which was almost equal to 3 h group. This result implied that 10% LV concentration might not be sufficient for transduction.

Compared to the microfluidic system, in the 96-well plate, the viral toxicity in 3-, 6-, and 12-hour groups was lower, where over 80% of the cells were alive 24 h post-infection. Of note, 10% LV concentration had no strong toxicity effect on the cells, regarding all time frames. However, in the 24 h group, 20% and 30% of LV concentrations significantly affected the cell survival in which only ∼56% and ∼20% of the cells remained alive 24 h post-infection. The considerable drop in the cell survival in the 24 h group compared to other groups betokened that in 96-well plates, viral particles need at least 24 h to affect the cells.

Overall, the toxic effects of LV particles were shown to be intensified in MFS in a shorter time period compared to 96-well plates. As mentioned above, the lower toxic effect in 96-well plate could be due to the waste of viral particles since the LVs move in random directions and the height of fluids in the well-plates are considerably higher than microfluidics (Fig. 1). The result of cell recovery and cell survival following infection indicated that to attain a proper protocol for our transduction system, a single round of infection with 20% LV concentration for 6 h in microfluidic could suffice for adequate transduction. However, since 6 h of infection in 96 well-plates might not result in proper transduction, we added the 24 h time period to our work, considering an equal amount of viral vectors used in microfluidic.

### 3.3 Transduction efficiency of LV-GFP in microfluidic

**Fig. 5:**
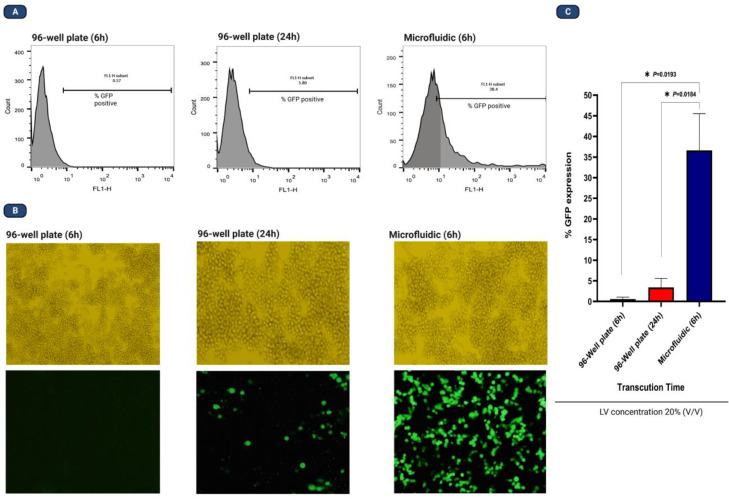
Enhancing LV transduction using a microfluidic system. A: flow cytometry analysis; B: invert microscopy; C: statistical analysis. Our analysis showcased that compared to 24 h in 96-well plates with 20% LV concentration, 6 h of infection in MFS can increase the transduction of NALM-6 cells by 9-folds. Based on flow cytometry results, 36.6 ± 8.8% of the NALM-6 cells from MFS expressed EGFP and only 3.42 ± 2.1% of the cells from the 96-well plate expressed EGFP.

After attaining a well-balanced cell recovery and cell survival setting, we compared 96-well plates with MFS for transduction efficiency. It should be mentioned that prior to detecting GFP in transduced groups, we assessed our batch of non-transduced NALM-6 cells for fluorescence emission. As was expected, there was no GFP-positive cell, ensuring that there was no false positive cell result. The established design used in our study was 6 h of infection using 20% LV concentration, and in parallel, we used the 96-well plate as the control group for MFS in order to assess the transduction efficiency. As was expected, transduction efficiency in the 6 h group of the 96-well plate showcased almost no GFP-positive cell, so we extended the infection time by adding a 24 h group. Six days post-transduction, GFP-positive cells were measured using flow cytometry and an invert fluorescence microscope (Fig. 5), where MFS outperformed the 96-well plate. The test was performed in triplicate, where the mean GFP expression in microfluidic was 36%, with the highest and lowest expression being 27% and 44%, respectively. The GFP expression was seen only in the 24 h group of 96 well-plates in the range of approximately 1% to 6%. Compared to the standard well-plate-based transduction, this data indicated that the microfluidic system is capable of enhancing the transduction efficiency using a single round of LV infection by almost 9-fold in relatively hard-to-transduce leukemic B-cell precursor cells (NALM-6).

## 4 Discussion

Here in this study, we have used a straightforward microfluidic-based approach to multiply the LVs transduction efficiency in BCP-ALL derived cells, called NALM-6. This approach can be extrapolated to other cell lines, as other studies have already done. Accordingly, a head-to-head analysis of transduction efficiency in MFS and 96 well-plates was performed, where the LV concentrations (v/v%), transduction times, cell survival, and surface-to-volume ratio for optimum transduction were evaluated. Based on the evidence of cell recovery and cell survival, we chose 6 h of incubation with 20% non-concentrated LV. Further, the result of EGFP expression showcased that a microfluidic system could increase the LV transduction of NALM-6 cells by almost 9-folds.

Our goal was to use a low volume of non-concentrated LVs to achieve a significant transduction rate of NALM-6 cells with low toxicity in less time compared to standard transaction protocols in well plates. Morgan J.R et al. have showcased that keeping the amount of LVs constant while decreasing the total transduction volume can increase the gene delivery efficiency [30]. Of note, they used standard well plates to reduce the volume to only 40 μL, while we minimized the transduction volume to 2 μL with MFS, keeping surface area equal to 96-well plates. Moreover, another study indicated that by increasing the infection time more viruses would be adsorbed and affect the cells in a logarithmic way [22], which is in accordance with our results from cell survival tests. Noteworthy, increasing the density of target cells by decreasing the total transduction volume is limited in standard cell culture systems, while MFS allows for much lower transduction volumes in a short transduction period before the environment becomes intolerable for cells. Increasing the density of cells by reducing total transduction volume in standard systems might cause high toxicity since the required transduction time is considerably higher. The transduction time is considerably lower in MFS, due to the fact that MFS can have a height around 50–70 μm compared to 96 well-plates (∼1400–1500 μm), which significantly increases the interaction of vectors and cells, providing a shorter transduction period. This allows a high number of cells to be transduced before the environment becomes nutrient-limited in a single round of transduction.

The chief mechanism responsible for promoting transduction efficiency in MFS is the enhancement of co-localization of viruses and target cells, increasing the probability of vector entry in a shorter amount of time. Virus entry has been considered a key rate-limiting step [31], and after the entrance, there is a multi-step process that depends on intrinsic factors regulating the target cells and the virus particles [24,32,33], though our goal was to mechanically juxtapose these two elements for a better transduction rate. Additional to MFS features, other mechanical and chemical manipulations can be measured to enhance the transduction rate [10,34,35]. Herein we used polybrene, which is a cationic agent facilitating the interaction of LVs ligand and cell receptors.

In a recent study by Tran and colleagues [27], they used various microfluidic designs to showcase that lowering the height of microfluidic channels can enhance the transduction efficiency in T-cells and primary hematopoietic stem and progenitor cells (HSPCs) up to 5-fold. This effect had also been seen in standard well-plates; as the height of the total volume decreased, the transduction efficiency increased. However, decreasing the total transduction volume is limited in standard well-plates. Their findings are greatly in accordance with our results in increasing the transduction rate in BCP-ALL cells, although they went further and coated their microfluidic devices with Retronectin, which could increase the transduction efficiency even more. It is noteworthy that Retronectin could significantly increase the transduction rate in well-plates, whereas MFS slightly benefits from Retronectin due to the high efficiency inherent in microfluidics [27].

Initial designs of microfluidics for enhancing viral transduction included a flow-through-based method, which could increase the transduction efficiency by around 2.75-fold higher than standard cell culture using low viral concentration. Albeit, increasing the viral concentration in this system wasted a considerable number of viral vectors since they had passed the membrane without contacting the target cells [35]. Herein, our design could achieve transduction efficiency by almost 9-fold; however, increasing the concentration of viral vectors does not lead to higher vector consumption. Recently, Moore and colleagues developed a more advanced MFS utilizing advective flow-through and a membrane in the middle of the device, which could efficiently bring the viruses to vectors. They increased the transduction efficiency in T-cells and hematopoietic stem cells (HSCs) by more than two-fold compared to standard well-plate controls using low viral concentration. It is worth mentioning that their flow-through system had a more sophisticated design and had significant advantages compared to our design, one of which is that they could manage and maintain the cell survival greatly and also significantly decrease the transduction time [36].

Luni et al. [37] presented a MFS for enhancing the efficiency of adenoviral transduction while working with low viral doses. Featuring 10 independent channels, the platform allows sequential perfusion stages that can be controlled automatically. Infection percentages and infecting virus distributions in the cell population were predicted using a stochastic mathematical model. In order to validate the system, human foreskin fibroblasts were infected with replication-incompetent adenoviruses carrying the EGFP gene. In contrast to a single infection at a higher viral dose, repeated pulses of cell-virus contact using the sequential microfluidic infection system enhanced exogenous gene expression at low doses. Silva and colleagues [38] used a more sophisticated approach for enhancing the adenoviral vectors transduction of pancreatic cells, in which they trapped the cells in microchambers. In accordance with Luni et al. study, the cells were immobilized and trapped alongside a constant flow of vectors upon target cells. The platform was validated using two different adenoviral vectors, resulting in transduction efficiencies of up to 98%. Our study aimed to enhance LV transduction efficiency in a straightforward approach. Compared to these two studies [37,38], we used a single round of infection without the need for cell coating and continuous media exchange to attain a high rate of transduction with minimal toxicity. In some cases, media exchange in microfluidics can result in losing cells. However, it should be mentioned that achieving a protocol that minimizes cell loss in systems such as the abovementioned studies greatly helps improve MFS-based viral transduction.

We observed that increasing incubation time in MFS after 6 h negatively affects cells and does not promote transduction efficiency. Two key factors can be mentioned as the underlying factors. First, nutrients in 2 μL of media for 100,000 cells could become drastically limited for the cells in time and virus particles can be toxic to the cells. Secondary to that, LV half-life is limited and after a time, it degrades [19,20]. Moreover, it is reported that when the virus reaches the cell, the fusion step starts within the first 5 minutes and the whole viral entrance process might take minutes to hours [39,40]. Overall, based on the evidence and design of MFS, 30 min to 6 h transduction is suitable [27,36,40].

The tests implemented throughout this study are centered around the utilization of LVs to showcase the features of MFS to enhance viral transduction. We believe that MFS is not limited to the LVs and can greatly help in optimizing the transduction efficiency of a wide range of viral vectors that are utilized in cell-based therapy market. Since the main mechanism of action for MFS is to co-localize the target cells and viral vectors in a micro-environment condition, its application can be extrapolated to other vectors as well. Additionally, the micro-scaled environment provided by MFS requires a considerably lower amount of material and reagents for the transduction procedure, which could help LV-based research be cost-effective.

## Ethics approval and consent to participate

Not applicable.

## Consent for publication

Not applicable.

## Availability of data and materials

Not applicable.

## Funding

None.

## Authors' contributions

MS conceived, edited, and revised the manuscript; S.E.A., R.M.S., and A.A.K., carried out the tests and experiments. S.E.A wrote the manuscript and prepared the tables and figures. All authors read and approved the final manuscript.

## Declaration of Competing Interest

The authors declare no competing interests.

## Data Availability

Data will be made available on request.

## Appendix Supplementary materials

Image, video 1
